# System accuracy evaluation of the new blood glucose monitoring meter “GLUCOCARD S onyx” beyond ISO 15197:2013/EN ISO 15197:2015 requirements and with new patient safety features

**DOI:** 10.3389/fcdhc.2025.1465732

**Published:** 2025-04-03

**Authors:** Daisuke Azuma, Hisashi Okuda, Beate Saeger

**Affiliations:** ^1^ Azuma Diabetes Clinic, Hyogo, Japan; ^2^ Scientific Activity Support Team, Kyoto Laboratory, ARKRAY Inc., Kyoto, Japan; ^3^ Scientific Affairs Division, ARKRAY Europe B.V., Amstelveen, Netherlands

**Keywords:** ISO 15197:2013, EN ISO 15197:2015, GLUCOCARD S onyx, blood glucose meter, YSI 2300, accuracy evaluation, Clark error grid, MARD

## Abstract

**Introduction:**

Blood glucose monitoring meters (BGM) have not become redundant yet. The accuracy and precision of “GLUCOCARD S onyx,” a new BGM with Bluetooth function, has been evaluated and proven to exceed the actual ISO 15197:2013/EN ISO 15197:2015 guidelines besides offering features for better patient safety and telemedicine.

**Methods:**

100 finger-prick whole blood samples from subjects with diabetes and 32 without diabetes were collected and measured with GLUCOCARD S onyx. Plasma blood glucose levels were measured using YSI2300 STAT PLUS as reference analyzer for comparison. The evaluation followed ISO 15197:2013, section 6.3 accuracy criteria. Furthermore, the MARD factor was calculated for the overall clinical important range (with n=132 samples).

**Results:**

The performance of GLUCOCARD S onyx was evaluated according to ISO 15197:2013, revealing that 99.7% (598/600) of the results fell within ±15% or ±0.8 mmol/L (± 15 mg/dL) of difference over the total clinically relevant glucose range compared to the YSI2300 STAT PLUS. 100% (600/600) of the measurement results over the total range fell within Clark Error Grid Zone A. An overall mean absolute relative difference (MARD) factor of 4.15% was obtained; 5.05% for glucose <5.6 mmol/L (<100 mg/dL), and 3.65% for glucose ≥5.6 mmol/L (≥100 mg/dL).

**Discussion:**

GLUCOCARD S onyx shows clinically satisfactory accuracy and reliability, even exceeding the ISO 15197:2013 criteria, for hypoglycemic cases with glucose critically low as <3.9 mmol/L (<70 mg/dL) and hyperglycemic cases with glucose ≥10.0 mmol/L (≥180 mg/dL). Healthcare organizations as well as manufacturers are aiming to offer new BGM systems that go beyond the ISO criteria and offer systems that can be consulted instead or besides CGM (Continuous Glucose Monitoring) in case of e.g. severe hypo- and/or hyperglycemic episodes. A MARD factor of 4.15% revealed an excellent system accuracy over the total clinically relevant glucose range. With additional user-friendly features, this BGM can be seen as a useful tool for efficient diabetes therapy, especially in the event of severe blood glucose fluctuations.

## Introduction

Many types of self-monitoring blood glucose meters (e.g., SMBG and BGM), as well as continuous glucose monitoring (e.g., CGM) devices, are available in the market to attain glycemic targets.

However, blood glucose meters have not become redundant or obsolete. They are still a reliable tool to reduce the risk of diabetes-related complications (1, 2).

More recent trials support the positive effect of strict blood glucose control (3).

A high analytical quality of a blood glucose monitoring system meeting defined standards, is crucial ensuring efficient diabetes therapy, especially in the case of severe blood glucose fluctuations, hypoglycemia suspect, avoiding diabetes shock situation and other incidents, such as fasting events for >8 hrs., sickness or starting a new medicine (4, 5).

The new generation of blood glucose meters is offering a variety of convenient and safe features for better patient care. The ISO 15197:2013/EN ISO 151097:2015 (International Organization for Standardization, herein called “ISO 15197”) guidelines have been established to set up rules to agree on ‘what is acceptable performance for BGMs’. The minimum accuracy performance criteria are 95% of the system’s results (>100 samples measured in duplicate across 3 different test strip lots) shall fall within either ±15 mg/dL of the average measured values of the reference method at blood glucose (BG) concentrations <100 mg/dL or within ±15% for BG concentrations ≥100 mg/dL. In addition, 99% of the individual BG results shall fall within zones A and B of the consensus error grid (6).

In this system accuracy study, we demonstrate that the new GLUCOCARD S onyx even exceeds the criteria set by ISO 15197 and can therefore be a valuable tool for diabetes patients with or without any CGM system to judge and manage critical episodes. Patients who are suspected of being experiencing hypoglycemic or hyperglycemic shock are better advised to use a precise blood glucose meter in an acute case than a CGM, which reacts with a time lag.

BGMs like the Glucocard S onyx can also offer valuable and convenient features to patients, such as hypo- and hyperglycemic alerts, detection of insufficient blood volume, and data transmission via a secured Bluetooth to a dedicated diabetes application on a mobile device or via a micro-USB cable to a supplied diabetes software, for easier data management and communication with a diabetes expert (7).

## Materials and methods

Study procedures were based on the ISO 15197 (6) requiring at least 100 capillary blood samples and testing with 3 different test strip lots. In this accuracy study 132 fingerprick capillary blood samples were obtained, 100 subjects with diabetes and 32 subjects without diabetes using six GLUCOCARD S onyx meters (ARKRAY Inc.; hereinafter referred to as “S onyx”) and three different GLUCOCARD S test strip lots (ARKRAY Inc.; hereinafter referred to as “test strips”). Measurements were performed in duplicate. The meters were set up according to the manufacturer’s instructions for use. The proper functioning of each meter was ensured using the manufacturer’s control solution (3 levels) before testing and at the end of each day.

A total of 300 µl of capillary whole blood was collected from the fingertip of the 100 diabetic subjects using BD Microtainer Safety Lancet (Becton, Dickinson & Co.) and transferred to a BD Microtainer tube (Becton, Dickinson & Co.) containing lithium heparin. Each time, 20 μL of the collected blood specimen was dropped onto a parafilm sheet, and measurements were taken in duplicate with two S onyx units and one test strip lot.

Subsequently, hematocrit levels (hereinafter referred to as “HCT”) were measured using the epoc2 blood gas analyzer (Siemens Healthineers AG). HCT values, pO2, humidity and temperature were tested to be within manufacturer’s specifications (ARKRAY Inc.). Samples outside the HCT range of 33 - 55% were excluded from this study.

The remaining blood was centrifuged to separate the plasma, which was measured with the YSI 2300 STAT PLUS Glucose and Lactate reference analyzer (Yellow Springs Instruments, hereinafter referred to as “YSI”). The YSI reference analyzer was calibrated with a NIST SRM-917 (NIST National Institute of Standards and Technology, SRM Standard Reference Material) traceable glucose standard solution.

The stability of the glucose concentration was assessed by calculating the difference between comparative blood samples taken before and after the measurements with the S onyx meters.

To cover the full clinically relevant glucose range of 2.8 mmol/L (50 mg/dL) to >22.2 mmol/L (>400 mg/dL), 32 nondiabetic fingertip samples were either spiked by adding high D-glucose concentration or kept at 37°C for approximately three to five hours to induce glycolysis and obtain low-concentration samples. The oxygen partial pressure in these modified samples was checked to assure that it was comparable to that of the unmodified samples.

## Results

**Figure 1:**
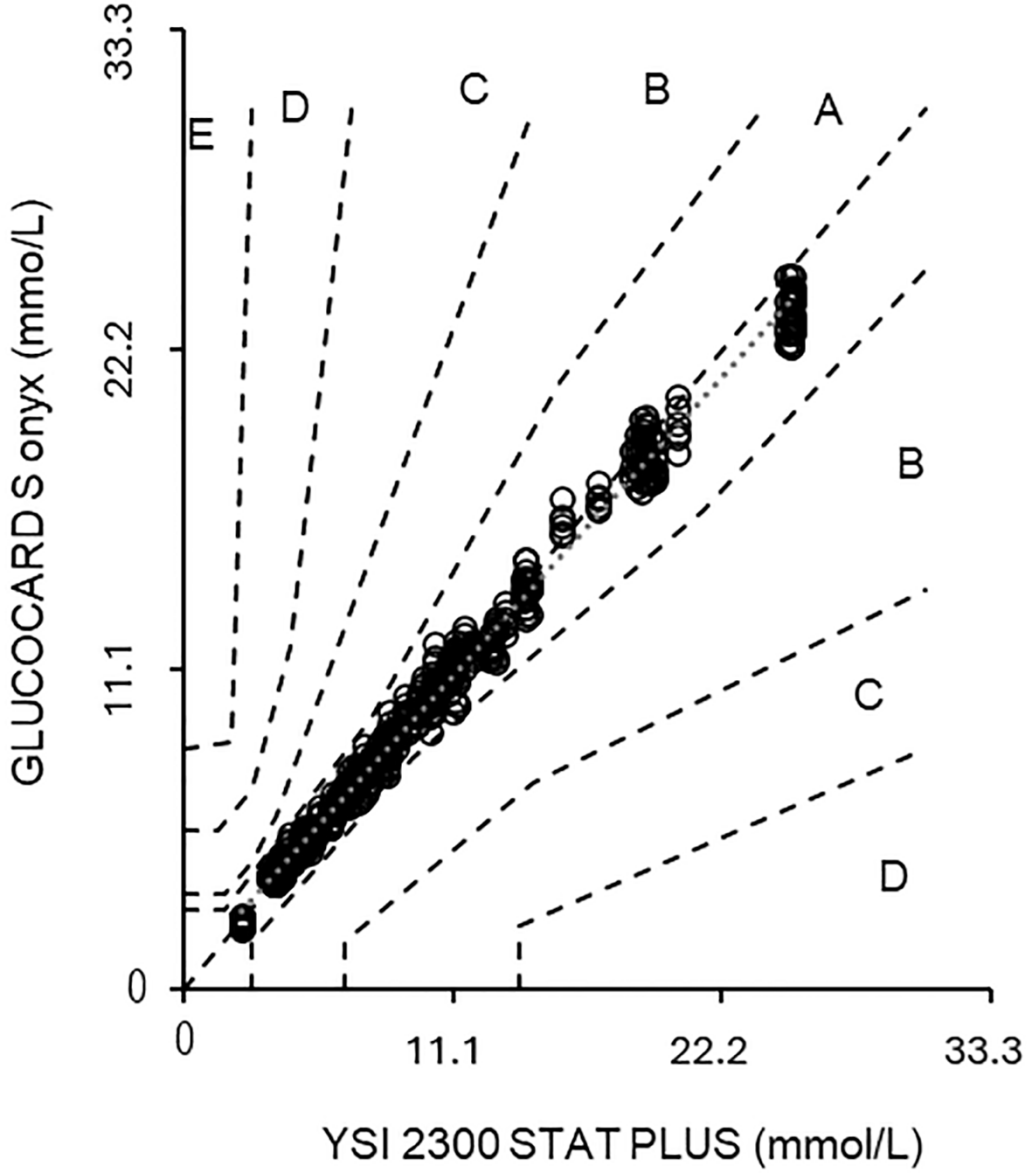
Shows the parkes consensus error grid graph (11), referring to YSI results. The y-axis shows the result of the S onyx, while the x-axis depicts the result obtained with the YSI. The zones within each area **(A–E)** illustrate the increasing clinical importance of an inaccurate measurement.

The GLUCOCARD S onyx meets the accuracy criteria of ISO 15197 with 97.5% to 100% of the results of each test strip lot within ±0.8 mmol/L (± 15 mg/dL) or ±15% of the results of the comparative method (**Table 1**) and 100% of the results in the CEG (Consensus Error Grid) A zone for all three lots (**Figure 1**).

**Table 1:** Conformity to minimum system accuracy performance criteria of ISO 15197.

Glucose concentration <5.6 mmol/L (< 100 mg/dL)					
within ±0.3 mmol/L (± 5 mg/dL)		within ±0.6 mmol/L (± 10 mg/dL)		within ±0.8 mmol/L (± 15 mg/dL)	
144/180	(80.0%)	173/180	(96.1%)	179/180	(99.4%)
Glucose concentration ≥5.6 mmol/L (≥100mg/dL)					
within ±5%		within ±10%		within ±15%	
291/420	(69.3%)	400/420	(95.2%)	419/420	(99.8%)
				Total range	
				within ±0.8 mmol/L (± 15 mg/dL) or ±15%	
				598/600	(99.7%)

The conformity to the ISO 15197 is shown in **Table 1**.

99.7% (598/600) of the measurement results fell within ±15% or ±0.8 mmol/L (± 15 mg/dL) over the total clinically tested glucose range, meeting the ISO 15197 system accuracy performance criteria (goal: >97.5 – 100%).

99.4% (179/180) fell within ±0.8 mmol/L (± 15 mg/dL) for glucose <5.6 mmol/L (<100 mg/dL).

99.8% (419/420) fell within ±15% for glucose ≥5.6 mmol/L (≥100 mg/dL).

Examining each lot of test strips, it was found that 99.0% (198/200) of Lot 1 samples, 100% (200/200) of Lot 2 samples, and 100% (200/200) of Lot 3 samples fell within the ISO 15197 performance criteria.

Furthermore, **Table 1**. revealed that even stricter criteria were met:

80% (144/180) fell within ±0.3 mmol/L (± 5 mg/dL) for hypoglycemia cases <5.6 mmol/L (<100 mg/dL) at a HCT of 42% (goal: accuracy >60%).

95.2% (400/420) fell within ±10% for hyperglycemia ≥5.6 mmol/L (≥100 mg/dL) at a Hct of 42% (goal: accuracy >60%).

Moreover, 90% of accuracy (65/72) of values fall within ±0.3 mmol/L (± 5 mg/dL) for severe hypoglycemia cases <3.9 mmol/L (<70 mg/dL) (goal: accuracy >60%); and 92% of accuracy (199/216) of values fall within ±10% for more severe hyperglycemia ≥10.0 mmol/L (≥180 mg/dL) (goal: accuracy >60%).

100% (600/600) of the GLUCOCARD S onyx data fell within the most accurate error grid zone A (“no risk zone”). The set ISO 15197 goal states that >99% shall fall within the zones A and B.

The regression equation against YSI was y = 0.94 x + 0.44.

The correlation coefficient was r = 0.996.

The MARD (Mean Absolute Relative Difference) methodology represents another way to measure device accuracy. The MARD calculates the average difference between a device test result and the reference measurement at normal to high glucose levels.

The MARD is calculated from the sum of |(BG meter)-(BG reference)|/(BG reference) measurements, divided by the number of measurements and is then multiplied by 100 (%).

The lower the MARD value (in %), the better the correlation between the device and the reference value or comparator measurement. A higher MARD value is an indication of a greater discrepancy between the measured reading and the reference result (**Table 2**).

**Table 2:** MARD calculation of S onyx vs. YSI (n=132).

MARD (Total)	4.15%
MARD < 5.6 mmol/L (<100 mg/dL)	5.05%
MARD ≥ 5.6 mmol/L (≥100 mg/dL)	3.65%
MARD ≥ 10.0 mmol/L (≥180 mg/dL)	3.37%

S onyx resulted in an overall MARD of 4.15%.

For glucose <5.6 mmol/L (<100 mg/dL) a MARD of 5.05% has been achieved.

For glucose ≥5.6 mmol/L (≥100 mg/dL), a MARD of 3.65% has been obtained.

For glucose ≥10.0 mmol/L (≥180 mg/dL), a MARD of 3.37% has been obtained.

## Discussion

In this study, the analytical performance of the GLUCOCARD S onyx was assessed based on ISO 15197. The system accuracy defines how well the measurement results of a system match the glucose level determined in parallel with a comparative method of a higher metrological order (2). Inaccurate results can influence therapeutic decision-making regarding insulin dosage and can therefore be of clinical relevance while worsening hypo- or hyperglycemia (8, 9). Easy Bluetooth data transfer to a mobile device and creating reports that can be shared with the diabetes doctor or nurse e.g. by email in case of an emergency can further accelerate a decision-making process.

The results have shown that S onyx complied well with the accuracy criteria of ISO 15197 for all the tested meters and reagent lots. Even stricter criteria for hypo- and hyperglycemic events were all met. Additionally, the MARD calculation revealed an excellent system accuracy of <5% across the total clinically relevant glucose range for S onyx (10).

In the CEG analysis (11), the results were distributed into five different risk zones (12–15), which showed that 100% of results from the S onyx fall into the clinically acceptable zone A. Considering the lot-to-lot variations that are an essential factor considering the accuracy of BGMs, it is of utmost importance to evaluate any released test strip lot in a harmonized manner to ensure compliance with established standards.

As BGMs continue to be more accessible and affordable than CGMs, choosing a BGM that combines proven accuracy with latest technological innovations will still make patients’ daily routines easier (16) and allow to react immediately in case of fast blood glucose fluctuations.

## Conclusions

The GLUCOCARD S onyx can be considered a precise and accurate new blood glucose monitoring system that offers not only superior performance (5, 6, 9) beyond the ISO 15197 guidelines but also useful safety features such as auto-coding for test strips and alerts for hypo- and hyperglycemia measurements and telemedicine. This would enable patients to manage their diabetes with confidence and healthcare providers to treat diabetes in even complex clinical scenarios with severe hypo- and hyperglycemic episodes. Additionally, it features specific flags, such as meal and bedtime markers; weekly and up to 90-day average calculations; newly added secured Bluetooth function or the use of a mini-USB cable for data transfer. Those features enable improved documentation and data management of blood glucose results through a mobile phone application or computer software tool, including consultations and report sharing with a family member or diabetes expert. For further consultation the manufacturer’s test strip IFU and meter user manual gives valuable additional information on possible common drug interferences or limitations.

## Data Availability

The original contributions presented in the study are included in the article/supplementary material. Further inquiries can be directed to the corresponding author.
